# HPRep: Quantifying Reproducibility in HiChIP and PLAC-Seq Datasets

**DOI:** 10.3390/cimb43020082

**Published:** 2021-09-17

**Authors:** Jonathan D. Rosen, Yuchen Yang, Armen Abnousi, Jiawen Chen, Michael Song, Ian R. Jones, Yin Shen, Ming Hu, Yun Li

**Affiliations:** 1 Department of Biostatistics, University of North Carolina, Chapel Hill, NC 27516, USA; jdrosen@live.unc.edu (J.D.R.); jiawenn@email.unc.edu (J.C.); 2 Department of Genetics, University of North Carolina, Chapel Hill, NC 26514, USA; yyuchen@email.unc.edu; 3 Department of Quantitative Health Sciences, Lerner Research Institute, Cleveland Clinic Foundation, Cleveland, OH 44195, USA; a.abnousi@gmail.com (A.A.); hum@ccf.org (M.H.); 4 Institute for Human Genetics, University of California, San Francisco, CA 94143, USA; song.michael12@gmail.com (M.S.); Ian.Jones3@ucsf.edu (I.R.J.); Yin.Shen@ucsf.edu (Y.S.); 5 Department of Neurology, University of California, San Francisco, CA 94143, USA

**Keywords:** reproducibility, HiChIP, PLAC-Seq, chromatin spatial organization

## Abstract

HiChIP and PLAC-Seq are emerging technologies for studying genome-wide long-range chromatin interactions mediated by the protein of interest, enabling more sensitive and cost-efficient interrogation of protein-centric chromatin conformation. However, due to the unbalanced read distribution introduced by protein immunoprecipitation, existing reproducibility measures developed for Hi-C data are not appropriate for the analysis of HiChIP and PLAC-Seq data. Here, we present HPRep, a stratified and weighted correlation metric derived from normalized contact counts, to quantify reproducibility in HiChIP and PLAC-Seq data. We applied HPRep to multiple real datasets and demonstrate that HPRep outperforms existing reproducibility measures developed for Hi-C data. Specifically, we applied HPRep to H3K4me3 PLAC-Seq data from mouse embryonic stem cells and mouse brain tissues as well as H3K27ac HiChIP data from human lymphoblastoid cell line GM12878 and leukemia cell line K562, showing that HPRep can more clearly separate among pseudo-replicates, real replicates, and non-replicates. Furthermore, in an H3K4me3 PLAC-Seq dataset consisting of 11 samples from four human brain cell types, HPRep demonstrated the expected clustering of data that could not be achieved by existing methods developed for Hi-C data, highlighting the need for a reproducibility metric tailored to HiChIP and PLAC-Seq data.

## 1. Introduction

Chromatin spatial organization plays a critical role in genome structure and transcriptional regulation [1,2,3]. During the last decade, great strides have been made in the mapping of long-range chromatin interactions, thanks to the rapid development of chromatin conformation capture (3C) based technologies. Among them, Hi-C enables genome-wide measurement of chromatin spatial organization [4,5] and has been widely used in practice. To ensure scientific rigor, various methods have been developed to assess the reproducibility of Hi-C data [6,7,8,9,10]. For example, HiCRep [6] first performs 2D smoothing to reduce the stochastic noise resulting from the sparsity of Hi-C data, and then quantifies reproducibility by calculating a stratified correlation, which is a weighted average of correlation coefficients between contact frequencies across specific one-dimensional (1D) genomic distance bands. HiC-Spector [8] adopts a different approach, transforming symmetric Hi-C contact matrices to their corresponding Laplacian matrices and then calculating similarity as the average of the distances between normalized eigenvectors. Similar to HiCRep, GenomeDISCO [7] relies on data smoothing, which is performed over a range of steps of the random walk to determine an optimal separation between biological replicates and non-replicates as measured by area under the precision–recall curve. The reproducibility measure is a function of distances between two contact matrices smoothed using this optimized number of steps. QuASAR-Rep [9] determines a local correlation matrix by comparing observed interaction counts to background signal–distance values within a specified distance. This local correlation matrix is subsequently transformed by element-wise multiplication with a matrix of scaled interaction counts. The reproducibility between two samples is defined as the Pearson correlation coefficient between the corresponding transformed matrices.

Recently, HiChIP [11] and PLAC-Seq [12] technologies (hereafter collectively referred to as HP for brevity) have been developed to study protein-mediated long-range chromatin interactions at a much reduced cost and greatly enhanced resolution relative to Hi-C. While the chromatin immunoprecipitation (ChIP) step involved in HP technologies allows for the cost and resolution benefits, it also introduces additional layers of systematic biases, which make analysis methods developed for Hi-C data potentially unsuitable for HP data.

To fill in this gap, we propose a novel method, HPRep, to measure the similarity or reproducibility between two HP datasets. HPRep is motivated by HiCRep [6], the previously described method developed for quantifying reproducibility of Hi-C data. Similar to HiCRep, HPRep leverages the dependence of chromatin contact frequency on 1D genomic distance; however, in contrast, HPRep models different ChIP enrichment levels (Section 2.1.2), which contribute to the systematic biases specific to HP data, and also incorporates an unbalanced data matrix that addresses the targeted structure of HP data in comparison to Hi-C data.

## 2. Materials and Methods

### 2.1. Details for HPRep Method

#### 2.1.1. Step 1

During the pre-processing step, intra-chromosomal reads are split into two groups: short-range reads (≤1 Kb) and long-range reads (>1 Kb). The short-range reads are used as a measure of ChIP efficiency in the regression framework described later in the pipeline. Long-range reads are used to determine long-range interactions, which are extracted and classified as either AND, XOR, or NOT sets based on whether 2, 1, or 0 (respectively) read ends overlap with a ChIP-Seq identified peak for the protein of interest. Additional details can be found in the MAPS paper [13].

#### 2.1.2. Step 2

The regression and normalization follow a multi-step procedure:

1. We modeled the non-zero intra-chromosomal contacts as a zero-truncated Poisson model with mean *µ_ij_.* The covariates for effective fragment length (FL), GC content (GC), mappability (MS), and ChIP enrichment level (IP) are provided by the feather pre-processing step (as implemented in the MAPS pipeline), and represent log(*x_i_* × *x_j_*), where *x_i_* and *x_j_* are the corresponding covariate for bin *i* and *j*, respectively. We fit regression models for the AND and XOR sets separately.

$$\log\left(\mu_{ij}\right)=\beta_{0}+\beta_{1}\cdot FL_{ij}+\beta_{2}\cdot GC_{ij}+\beta_{3}\cdot MS_{ij}+\beta_{4}\cdot IP_{ij}$$

2. Fitted values were determined for each bin pair based on the resulting model for AND and XOR sets in each chromosome, resulting in 2 × *n* files where *n* is the number of autosomal chromosomes. In addition, the AIC and BIC values for each fitted model are supplied in a single file.
3. Normalized values are defined as log_2_(1 + observed/fitted) and all bin pairs are combined into one file. Additionally, the ChIP-Seq peaks are binned to analysis resolution and supplied as a file containing a list of these anchor bins. Peaks that span a bin boundary are assigned to all bins they span.

#### 2.1.3. Step 3

The final step involves data smoothing and sample comparison to calculate a final reproducibility metric between each pair of samples as a weighted Pearson correlation. The combined AND and XOR normalized data are stored in a matrix that is used as an input for the comparison algorithm. The basic data structure we considered was an *N* × *m* matrix, where *N* represents the number of anchor bins in the union set of anchors from all samples and *m* is equal to 2 × binning distance/resolution, where binning distance is recommended to be set at 1 Mb, but can be user specified. Interactions further than 1 Mb are typically sparse and highly variable. The *ij* element of the matrix represents the normalized contact frequency between the anchor *i* and the bin *j* bin widths away, *j* ∈ {−*m*/2, …, −1, 1, …, *m*/2}. For example, at a recommended binning distance of 1 Mb, *m* = 400 at 5 Kb resolution, and 200 at 10 Kb resolution.

The normalized values undergo a 1D smoothing procedure as follows: for a specified window size *d*, the *ij* element (*x_ij_*) is transformed so that the smoothed value is

(1) $$x_{ij}^{smoothed}=\left(\overset{j+d}{\underset{k=j-d}{\sum }}x_{ik}\right)/(2d+1)$$

Let *a_k_* and *b_k_* be two vectors of length 2*N* from samples *a* and *b*, respectively, whose elements consist of the values from the smoothed data matrix from columns ±*k* units symmetrically from the center. All these values represent normalized and smoothed contacts that are ±*k* bins from their respective anchors. Let *a_k_′* and *b_k_′* be the resulting vectors of length *N_k_* ≤ 2*N* after removing any elements satisfying *a_i_′* = *b_i_′* = 0, where *a′_ki_* is the *i*th element of vector *a_k_′*. We define *r_k_* as

(2) $$r_{k}=\frac{N_{k}\sum_{i=1}^{N_{k}}a_{i}^{’}b_{i\;}^{’}-\sum_{i=1}^{N_{k}}a_{i}^{’}\;\sum_{i=1}^{N_{k}}b_{i}^{’}\;\;\;}{\sqrt{N_{k}\sum_{i=1}^{N_{k}}a_{i}^{’2}-{\left(\sum_{i=1}^{N_{k}}a_{i}^{’}\right)}^{2}}\sqrt{N_{k}\sum_{i=1}^{N_{k}}b_{i}^{’2}-{\left(\sum_{i=1}^{N_{k}}b_{i}^{’}\right)}^{2}}}$$

namely the empirical correlation between *a′_k_* and *b′_k_*. They define the weights for each of the *k* strata as

(3) $$w_{k}=\frac{N_{k}\;\sqrt{\frac{\sum_{i=1}^{N_{k}}a_{i}^{'2}}{N_{k}}-{\left(\frac{\sum_{i=1}^{N_{k}}a_{i}^{'}}{N_{k}}\right)}^{2}}\sqrt{\frac{\sum_{i=1}^{N_{k}}b_{i}^{'2}}{N_{k}}-{\left(\frac{\sum_{i=1}^{N_{k}}b_{i}^{'}}{N_{k}}\right)}^{2}}}{\overset{K}{\underset{k=1}{\sum }}\;N_{k}(\sqrt{\frac{\sum_{i=1}^{N_{k}}a_{i}^{'2}}{N_{k}}-{\left(\frac{\sum_{i=1}^{N_{k}}a_{i}^{'}}{N_{k}}\right)}^{2}}\sqrt{\frac{\sum_{i=1}^{N_{k}}b_{i}^{'2}}{N_{k}}-{\left(\frac{\sum_{i=1}^{N_{k}}b_{i}^{'}}{N_{k}}\right)}^{2}}}$$

The reproducibility score between two matrices is then the weighted average of the stratified correlations *r_k_*

(4) $$reproducibility\;score=\overset{K}{\underset{k=1}{\sum }}r_{k}w_{k}$$

### 2.2. Smoothing Parameter Optimization

The smoothing parameter *d* (Equation (1)) was tuned using the method similar to the HiCRep protocol with modification to the sampling scheme and search termination criterion. The following algorithm was used:

Two samples to be analyzed were selected, preferably dissimilar ones such as non-biological replicates. Twenty-five percent of the non-zero contacts from one were randomly sampled and used to populate a contact matrix as previously diagrammed, with the remaining entries set to zero. The analogous positions in the other sample were used to populate a corresponding matrix. The reproducibility score was calculated for these matrices and the sampling procedure was repeated a total of ten times with no smoothing performed. The average of these ten values was recorded.

The smoothing parameter was then iterated by one, repeating the above procedure until the average metric using smoothing parameter *d* + 1 compared to *d* exhibited less than a one percent increase. The value of *d* was recorded and used as the smoothing parameter for all analyses with the particular dataset.

### 2.3. Procedures for Comparative Methods

#### 2.3.1. HCRep

All results obtained using HiCRep were conducted using R (3.6.0) and using version 1.12.0 of the HiCRep package obtained from https://github.com/MonkeyLB/hicrep (accessed on 29 April 2020). Default parameters were used for all experiments. Note that the documentation recommends a smoothing parameter of 20 for 10 kb resolution, but does not specify a recommended parameter for 5 kb resolution. We used 20 for 5 kb as well since marginal difference was reported when tuning beyond 20.

To ensure proper data formatting for use with HiCRep, the built-in function “bed2mat” was utilized, which converts a 3-column contact matrix to a square contact matrix with all elements not supplied set to 0. Experiments that included solely AND XOR sets of contacts were prepared by extracting bin pairs and observed (integer) contacts from the corresponding AND/XOR files and those that also included NOT sets were generated similarly.

#### 2.3.2. HiC-Spector

The Python version of HiC-Spector was used rather than the Julia version since the former readily accepts Hi-C data in genomic coordinates rather than the hic format. The program used was “run_reproducibility_v2.py” found at https://github.com/gersteinlab/HiC-spector (accessed on 17 February 2020). Experiments that included solely AND and XOR sets of contacts were prepared by extracting bin pairs and observed (integer) contacts from the corresponding AND/XOR files. Note that the bin positions had to be converted to indices starting at 1, so the global minimum bin position was determined, and all bin positions scaled by (genomic position—minimum position)/resolution. Experiments also including NOT sets were generated similarly.

#### 2.3.3. Pearson Correlation

The upper triangular component of a standard symmetric *n* × *n* contact matrix was flattened to a vector for each sample. The Pearson correlation between two samples was computed as the correlation between these vectors.

### 2.4. Down-Sampling Procedure

The generalized downsampling procedure was performed on the AND and XOR contact files for each chromosome separately. Let *n* be the total number of counts for all bin pairs in the specific file and let *d* be the downsampling coefficient. That is, to downsample to 0.8 × depth, *d* = 0.8. The vector *v* of counts for all bin pairs was downsampled to depth *d* utilizing the R function “rmultinom”, where the size parameter was set to floor (*n* × *d*) and the probability vector was the element-wise division of *v* by *n*. These downsampled AND and XOR files then intersected the pipeline as usual with the removal of bins that now have counts of 0.

### 2.5. Determination of Silhouette Values

Silhouette values were calculated via the method in [14]. Let *d*(*i, j*) be the similarity between sample *i* and *j*, which in this analysis was the scaled reproducibility metric between the two samples. The silhouette method requires that the similarity (or distance) quantities be comparable on a ratio scale, that is, if the distance between two points is doubled, it implies that the points are twice as far apart. The Pearson correlation does not have such a property, so for each experiment the values were standardized to [0, 1] by subtracting the lowest value and dividing by the (max − min) value.

Let sample *i* be a member of cluster *A*. Furthermore, let *a*(*i*) be the average similarity of *i* to all other samples in the same cluster. Let *d*(*i, C*) be the average similarity of sample *i* to all other samples in cluster *C* and let *b*(*i*) be the maximum value of *d*(*i, C*) over all clusters *C* distinct from cluster *A*. Then, the silhouette value is defined as

(5) $$s\left(i\right)=\frac{a\left(i\right)-b\left(i\right)}{max\left\{a\left(i\right),\;b\left(i\right)\right\}}$$

We report the average *s*(*i*) over all 11 samples. The closer this value is to 1, the better the clustering performance.

### 2.6. Data Details

For the human brain PLAC-Seq data, fastp (https://github.com/OpenGene/fastp (accessed on 11 February 2019) was used to trim the fastq files to 100 bp. No additional modifications to the described pipeline were performed on any of the datasets used in this paper. Default software options described in https://github.com/yunliUNC/HPRep (accessed on 16 October 2020) were used for alignment and merging for all samples analyzed. Resolutions used for each dataset were:

1. Mouse embryonic stem cell and mouse brain tissue H3K4me3 PLAC-seq: 10 Kb
2. Human brain H3K4me3 PLAC-seq:                  5 Kb
3. GM12878 and K562 H3K27ac HiChIP:                 10 Kb

### 2.7. Irreproducible Discovery Rate

ChIP-Seq data processing followed the procedure outlined in [13]. Specifically, MACS2 (v 2.1.2) was used to provide the narrowPeak input files using flags: --nolambda, --nomodel, --extsize 147, --call-summits, -B, --SPMR, and -q 1 × 10^−2^. These files were processed using IDR (v 2.0.4.2) with default parameters. Results reported represent the fraction of peaks that exceed a false discovery rate of 5%. Downsampling was performed on the MACS2 input files by randomly selecting an appropriately sized subset of reads.

## 3. Results

Currently available methods to quantify reproducibility in Hi-C datasets such as HiCRep, HiC-Spector, GenomeDISCO, and QuASAR-Rep (systematically evaluated in [10]), all involve derivation of a similarity metric between two contact frequency matrices. The input Hi-C data consists of *n* × *n* symmetric matrices of non-negative integers, where each row/column represents one genomic locus (i.e., bin) and *n* is the total number of bins. The (*i,j*) element of such a matrix represents the number of paired-end reads spanning between bin *i* and bin *j*. 

These existing methods are conceptually inappropriate for HP data due to the unbalanced read distribution due to ChIP enrichment that is introduced in the HP experiments. 

In addition, while Hi-C data consist of interactions among all bin pairs, HP data are restricted to bin pairs where at least one bin overlaps a binding region of the protein of interest. Such overlapping bins are referred to as the anchor bins, and two HP datasets may have different sets of anchor bins. We further define bin pairs consisting of two anchor bins as the “AND” pairs, and those consisting of only one anchor bin are defined as the “XOR” pairs. In contrast, the “NOT” pairs, for which neither bin is an anchor bin, are not meaningful due to the nature of HP technologies and therefore not used in HP data analysis [13].

**Figure 1:**
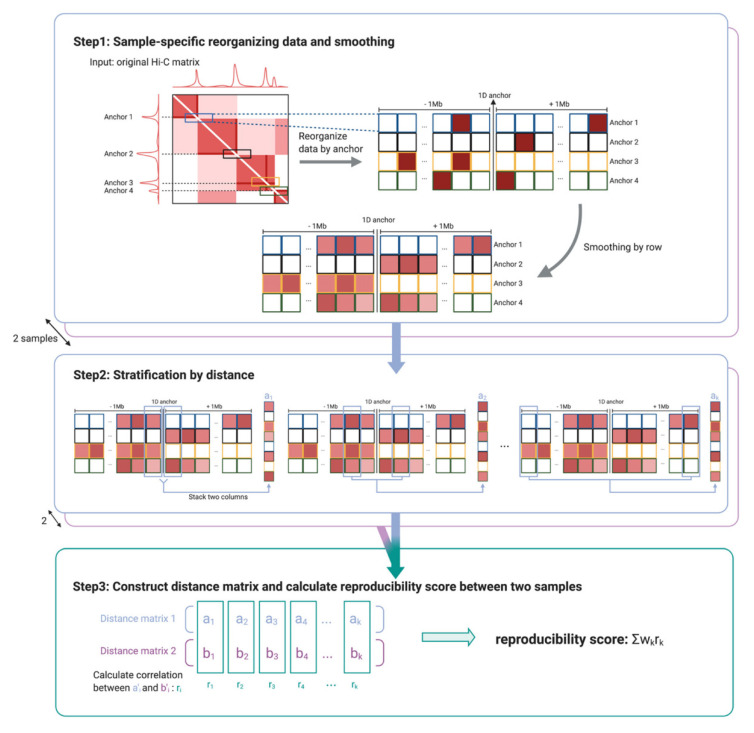
Cartoon illustration of HPRep. Step 1 involves first identifying anchors (i.e., 1D ChIP peak sites) and then extracting all interactions between these anchors and bins within a specified genomic distance from the anchors. This is followed by a one-dimensional smoothing procedure. Stratification by 1D genomic distance is performed in step 2 so that the elements of vector *a_k_* represent interactions that are equidistant from their respective anchors, *k* bins apart. In the final step, the Pearson correlation coefficients are calculated between vectors from two samples both of stratum *k*, repeated over all *k*, and these Pearson correlation coefficients were combined in a weighted average to yield the final reproducibility metric.

The data structure in HPRep is an *N* × *m* matrix (Figure 1), where *N* represents the number of anchor bins and *m* = 2 * 1 Mb/resolution, where resolution refers to the bin size (1 Mb is set as the default but can be modified by the user). The (*i,j*) th element represents the normalized contact frequency between anchor *i* and the bin *j* bin widths away from the anchor, *j* ∈ {−*m*/2, …,−1, 1, …,*m*/2}. The number of anchor bins, *N*, is the cardinality of the union set of anchor bins for all datasets in the study. Normalization is performed via a two-step procedure. (1) Raw counts are adjusted for the biases introduced by effective fragment length, GC content, mappability, and ChIP efficiency by fitting a positive Poisson regression model, following the approach detailed in the MAPS method [13]. Separate models are fit to the AND and XOR pairs since the AND pairs are expected to have significantly higher contact frequencies due to double ChIP enrichment. (2) Using the fitted models, the data are normalized by taking the log_2_ value of (1 + observed/expected counts). Further details can be found in Section 2.1.

Similar to HiCRep [6], the distance metric used by HPRep is a weighted Pearson correlation coefficient that is stratified by 1D genomic distance. Note in Figure 1 that these strata are the pairs of columns of the previously described data matrix, which are equal-distant from the center. Due to the sparsity of HP data, especially for long-range chromatin interactions, the normalized count values were smoothed. The smoothing procedure used was a 1D arithmetic mean of values within a window of *d* bins away along the same row (see Section 2.2 for optimization procedure). Each of the *m*/2 correlations was weighted based on the variation of the smoothed values at that distance such that the weights sum to one. Therefore, the resultant metric was restricted to [−1, 1] and had a similar interpretation as a standard Pearson correlation coefficient. 

Let *a_k_* and *b_k_* be two vectors of length 2*N* from samples a and *b*, respectively, whose elements are normalized contact counts, where *N* represents the number of anchor bins in the union set of anchor bins from all samples in the study, and *k* indexes bins that are ±*k* units away. Let *a_k_′* and *b_k_′* be the resulting vectors of length *N_k_* ≤ 2*N* after removing any elements that are 0 in identical positions in both two vectors. The weight for stratum *k*, *w_k_*, is defined as

(6) $$w_{k}=\frac{N_{k}\;\sqrt{\frac{\sum_{i=1}^{N_{k}}a_{i}^{'2}}{N_{k}}-{\left(\frac{\sum_{i=1}^{N_{k}}a_{i}^{'}}{N_{k}}\right)}^{2}}\sqrt{\frac{\sum_{i=1}^{N_{k}}b_{i}^{'2}}{N_{k}}-{\left(\frac{\sum_{i=1}^{N_{k}}b_{i}^{'}}{N_{k}}\right)}^{2}}}{\overset{K}{\underset{k=1}{\sum }}\;N_{k}\left(\sqrt{\frac{\sum_{i=1}^{N_{k}}a_{i}^{'2}}{N_{k}}-{\left(\frac{\sum_{i=1}^{N_{k}}a_{i}^{'}}{N_{k}}\right)}^{2}}\sqrt{\frac{\sum_{i=1}^{N_{k}}b_{i}^{'2}}{N_{k}}-{\left(\frac{\sum_{i=1}^{N_{k}}b_{i}^{'}}{N_{k}}\right)}^{2}}\right)}$$

where *K* is the total number of strata, which is analogous to the weights used in HiCRep [6]. The numerator of *w_k_* is the product of strata size and the standard deviations of *a_k_′* and *b_k_′*, while the denominator is the sum of the numerators over all strata. Consequently, the weights were restricted to [0, 1] and the sum to 1, where larger and more variable strata carry more weight than smaller and less variable strata. The final reproducibility metric was the weighted sum of correlations between each stratum. This workflow is diagrammed in Figure 1.

### 3.1. Mouse H3K4me3 PLAC-Seq Data

To evaluate the performance of HPRep, we first analyzed published H3K4me3 PLAC-Seq datasets from mouse embryonic stem cells (mESCs) [13] and mouse brain tissues [15], both consisting of two samples, by applying HPRep at 10 Kb resolution. Samples from the same cell type or tissue were labeled as biological replicates while those cross cell type or tissue were labeled non-replicates, yielding two pairs of biological replicates and four pairs of non-replicates. Pseudo replicates were generated by pooling two samples of the same cell type or tissue together, and then partitioning the pooled contact frequency in each bin pair randomly via binomial (*p* = 0.5) sampling.

**Figure 2:**
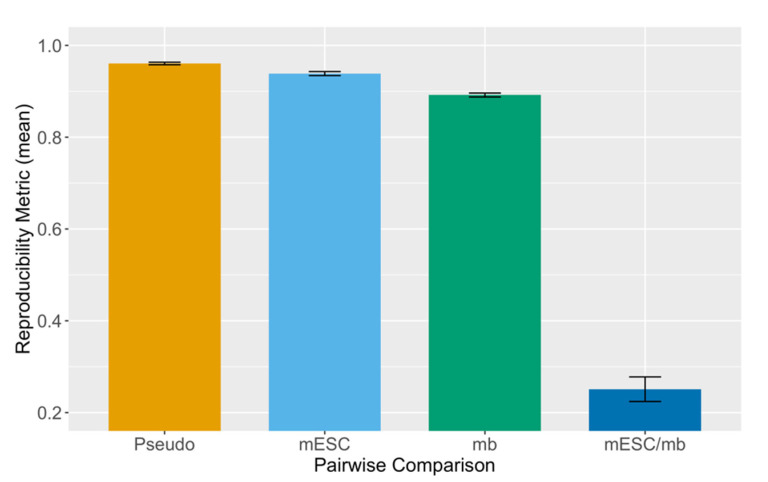
HPRep in mouse PLAC-Seq data. Metrics obtained applying HPRep to PLAC-Seq data from mESC and mouse brain (mb) tissues. Pseudo replicates generated from pooling two mESC samples followed by random sampling. Cross sample results represent the mean of four pairings. Results are presented as the mean value over 19 autosomal chromosomes with error bar representing ±1 standard deviation.

We would expect that pseudo replicates are most similar, followed by biological replicates, and that non-replicates are least similar. Indeed, this expected pattern is observed using HPRep (Figure 2), with results also exhibiting highly consistent patterns across chromosomes (Supplementary Figure S1). The higher metric for replicate mESC samples relative to mouse brain samples is due to the higher sampling depth of the former.

**Figure 3:**
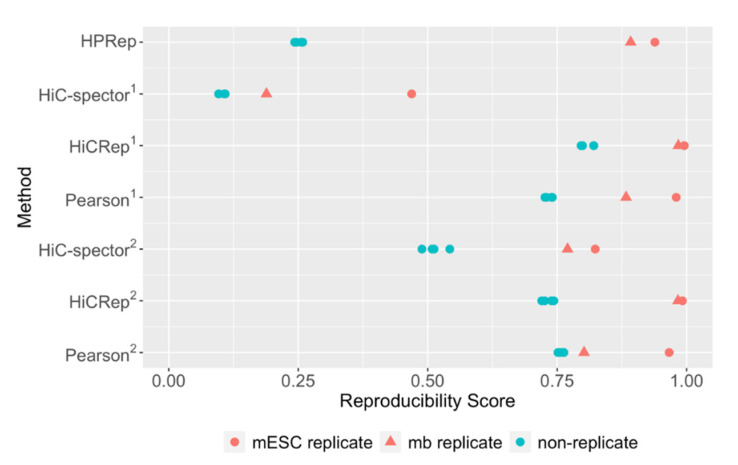
Comparison of methods in mouse PLAC-Seq datasets. HPRep compared to Hi-C specific methods HiC-Spector and HiCRep as well as Pearson correlation. (1) All methods using bin pairs in the AND and XOR sets. (2) Methods other than HPRep using all bin pairs in the AND, XOR and NOT sets. PLAC-Seq dataset consisted of two mESC and two mouse brain replicates.

We next compared HPRep with alternative methods, specifically two Hi-C reproducibility methods: HiCRep [6] and HiC-Spector [8] as well as a naïve Pearson correlation (Section 2.3). Since the Hi-C specific methods are designed using n × n symmetric contact matrices as the standard input, for these comparisons, in addition to restricting to bin pairs in the AND and XOR sets, we generated a “pseudo Hi-C” dataset from a HP dataset by also using all bin pairs (including the AND, XOR and NOT sets). The naïve Pearson correlation consisted simply of converting the entire upper triangular Hi-C contact matrices for each sample to single vectors and calculating the Pearson correlation coefficient between them. The methods were performed separately on all 19 autosomal chromosomes and the resulting metrics were reported as the arithmetic mean. The HiCRep and HiC-Spector methods were applied with the default parameters. The results are displayed in Figure 3.

All methods except for naïve Pearson correlation yielded results consistent with what we expected, namely higher similarity for the biological replicates and lower similarity for the non-replicates. The similarity or reproducibility values for the biological replicates were similar among these three methods, which is expected for HPRep and HiCRep, since both methods are based on stratified Pearson correlation, but is noteworthy for HiC-Spector, since it is based on a rather different method, and was restricted to [0, 1] as opposed to [−1, 1]. The difference among these methods, with the exclusion of HiC-Spector when including the NOT set, manifests largely in values for non-replicates, with HPRep yielding much smaller values relative to the others, although in each case, the four non-replicate pair results were very consistent. Interestingly, the naïve Pearson correlation fails with the mouse brain sample, yielding a reproducibility score nearly identical to those of the non-replicates, whereas the result from mESC replicates is consistent with the other three methods. This failure is obviated in HiCRep and HPRep, the other Pearson based methods. For example, for biological replicates, HPRep yields a mean reproducibility metric of 0.92 compared to a mean value of 0.25 for non-replicates. For the experiments using bin pairs in the AND, XOR and NOT sets, the mean reproducibility metrics comparing replicates and non-replicates were 0.80 vs. 0.51, 0.99 vs. 0.73, and 0.88 vs. 0.76 for HiC-Spector, HiCRep, and Pearson correlation coefficients, respectively.

### 3.2. Human HiChIP Data

**Figure 4:**
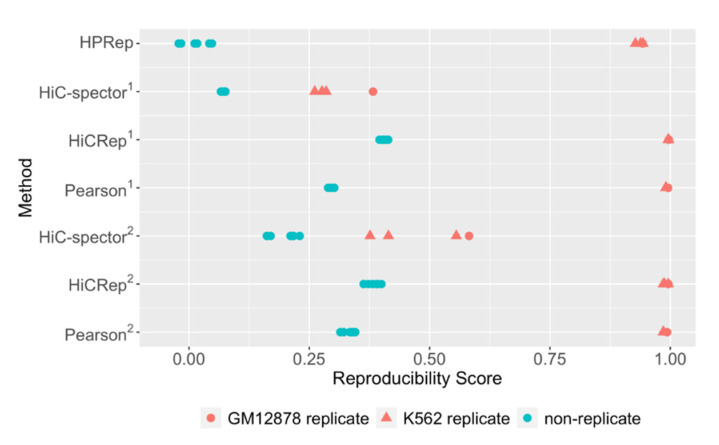
Comparison of methods in HiChIP datasets from human blood cell lines. HPRep compared to Hi-C specific methods HiC-Spector and HiCRep as well as Pearson correlation. (1) All methods using bin pairs in the AND and XOR sets. (2) Methods other than HPRep using all bin pairs in the AND, XOR, and NOT sets. HiChIP dataset consisted of two GM12878 replicates and three K562 replicates.

In addition, we applied HPRep to measure the reproducibility of H3K27ac HiChIP data from GM12878 cells (two biological replicates) and K562 cells (three biological replicates) at 10 Kb resolution [16], resulting in four pairs of biological replicates (one pair from GM12878, three pairs from K562) and six pairs of non-replicates (Figure 4). We anticipated a priori that differences between replicates and non-replicates would be more pronounced in this human dataset than the previous mouse H3K4me3 PLAC-Seq dataset due to the greater dissimilarity in H3K27ac anchor bins between GM12878 cells and K562 cells. Specifically, the GM12878 and K562 cell lines contained 31,980 and 26,963 H3K27ac 10 Kb anchor bins genome-wide (autosomal), respectively, with only 14,304 shared (Jaccard index 0.32). In contrast, mESC and mouse brain had 28,903 and 21,778 H3K4me3 10 Kb anchor bins, with 17,722 overlapping, (Jaccard index 0.54), which was expected since active promoters are largely shared across tissues and cell lines. For this human dataset, all methods were performed individually on all 22 autosomal chromosomes and the resulting metrics were averaged across chromosomes.

The results from the human HiChIP data were consistent with those from mouse PLAC-Seq data: the biological replicates yielded high similarity (close to 1) while the non-replicates yielded uniformly lower similarity. While all autosomal chromosomes were used in these analyses and the results were largely consistent across them using HPRep, HiCRep, and Pearson correlation coefficients, the results were quite inconsistent using HiC-Spector (Supplementary Figure S2). Specifically, HiC-Spector used 20 eigenvectors in the computation of a reproducibility metric, however, for several chromosomes, convergence failed, so fewer eigenvectors were used, which yielded erratic results (Supplementary Table S1). Again, HPRep results in the lowest metrics for the non-replicates, which were all close to zero, highlighting the influence on anchor bin identity in this method.

### 3.3. Human PLAC-Seq Data

**Figure 5:**
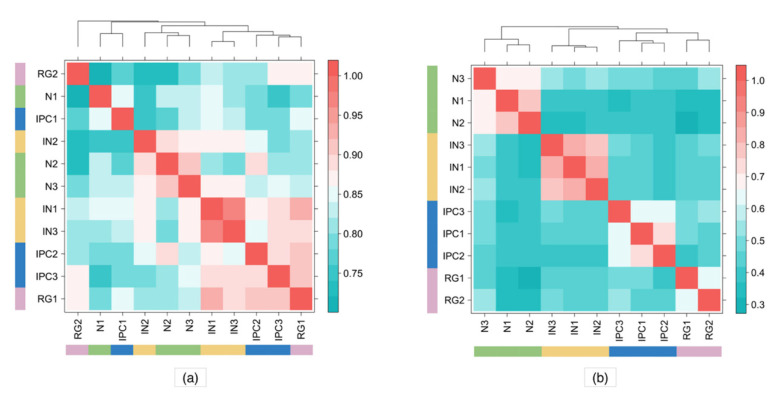
Comparison of HPRep and HiCRep in human brain PLAC-Seq datasets. (**a**) HiCRep and (**b**) HPRep. Dendrograms above the heatmaps indicate clustering determined by hclust function in R. HiChIP dataset consisted of three neurons, three interneurons, two radial glial, and three intermediate progenitor cell samples. Red color signifies results indicating stronger correlation.

We next applied HPRep to a more complex H3K4me3 PLAC-Seq dataset at 5 Kb resolution, consisting of 11 samples from four brain cell types in human fetal brain obtained via fluorescence-activated cell sorting [17]: three samples from neurons (N), three samples from interneurons (IN), two samples from radial glial (RG), and three samples from intermediate progenitor cells (IPC). These samples had varying sequencing depths (detailed in Supplementary Table S2 in [17]), with the number of intra-chromosomal reads ranging from 47.5 million for RG2 (the second replicate of RG) to 390 million for RG1 (the first replicate of RG). The anchor bins were defined as the union of 1D H3K4me3 peaks from all four cell types. In Figure 5a, reproducibility obtained by HiCRep showed no differentiation between inter- and intra-cell types. In contrast, HPRep showed a clear pattern of higher similarity for replicates from the same cell type compared to those from different cell types.

**Figure 6:**
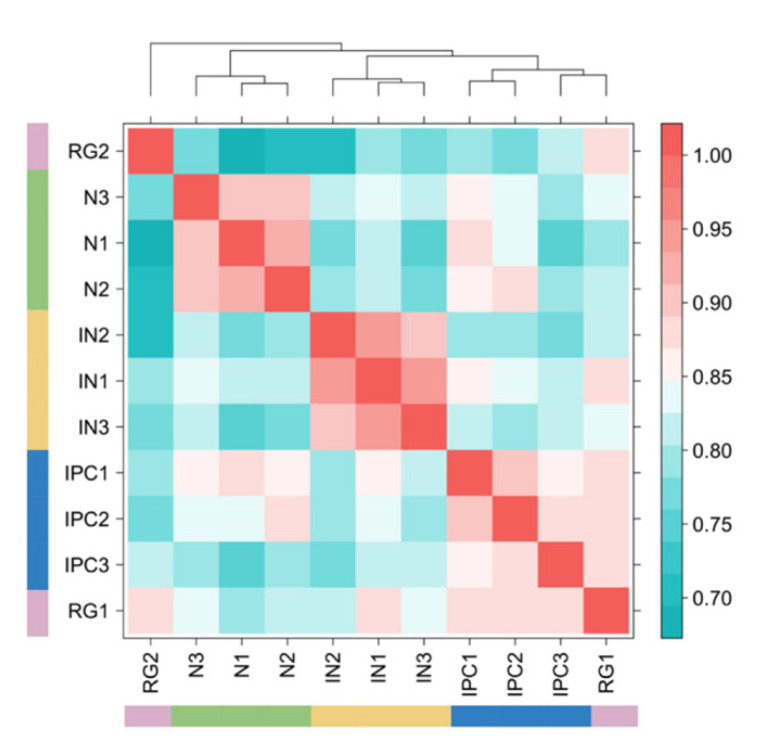
HiCRep excluding NOT pairs in human neural PLAC-Seq datasets. HiChIP dataset consisted of three neurons, three interneurons, two radial glial, and three intermediate progenitor cell samples excluding interactions where neither bin overlapped with an anchor. Red color signifies results indicating stronger correlation.

Focusing on bin pairs in the AND and XOR sets highlights the effect of normalizing ChIP enrichment level. Figure 6 is analogous to 5a excluding bin pairs in the NOT set. The cell type clustering is more in line with the known truth, however, still has misspecifications according to the dendrogram: neuron, interneuron, and IPC cells were correctly grouped, but radial glial cells were misclassified into two groups.

Recent studies have shown that HiCRep is sensitive to sequencing depth [10]. To evaluate the robustness of HPrep with respect to different sequencing depths, we performed downsampling to the original PLAC-Seq data from four human brain cell types. This was performed by sampling from a multinomial distribution with *n* equal to the original count multiplied by a downsampling factor and count probabilities set to match the distribution in the original data (Section 2.4).

**Figure 7:**
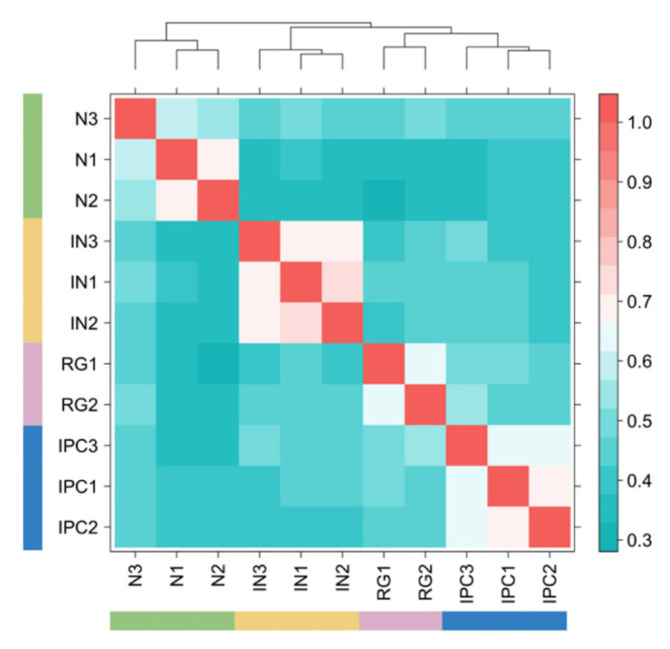
Performance of HPRep in downsampled human neural PLAC-Seq data. HPRep results obtained after downsampling all eleven samples to read depth of the lowest sample.

The first downsampling was performed so that all samples matched the depth of the sample (RG2), which had the lowest sequencing depth. Note the identical color scales for Figure 5b and Figure 7, but the decrease in metric values for many pairwise comparisons for samples of the same cell type such as interneuron cells. To quantify this reduced discernibility between samples, we utilized the silhouette procedure [14], treating reproducibility score as a distance metric and reporting the average of the 11 silhouette values, one for each sample (Section 2.5). We obtained 0.717 and 0.685 for the original experiment and downsampled results respectively, where smaller numbers indicate worse clustering performance.

**Figure 8:**
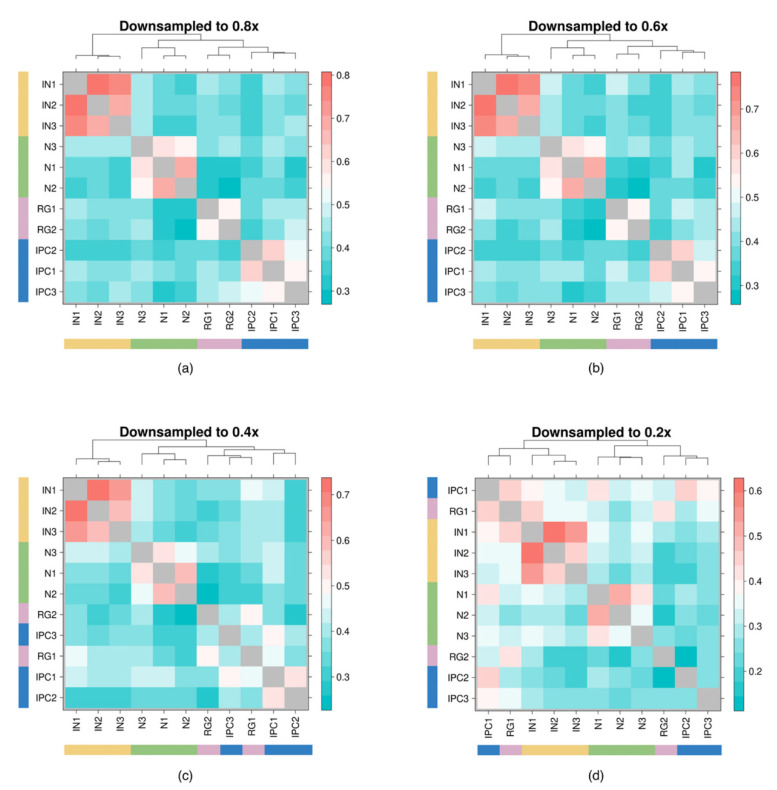
Downsampling uniformly across all samples in human neural PLAC-Seq datasets. HPRep results obtained after downsampling each sample by a specified factor: (**a**) 80% of original depth of each sample, (**b**) 60% of original depth, (**c**) 40% of original depth, (**d**) 20% of original depth. Note that the diagonal is now gray to remove it from the scaling to better highlight differences.

Subsequent downsampling was performed uniformly across all samples so that the total counts were reduced to 80%, 60%, 40%, and 20% of their original values following the previously described sampling protocol. As expected, in Figure 8, we observed decreased discernibility among samples from different cell types, most strikingly with IPC and RG where the within sample HPRep reproducibility metric dropped to as low as 0.26 and 0.43, respectively. Applying the modified silhouette procedure described above to these four downsampled datasets, we obtained a silhouette score of 0.700, 0.678, 0.634, and 0.518 for downsampling to 80%, 60%, 40%, and 20%, respectively.

We next sought to investigate the extent to which our HPRep metric was driven by the 1D ChIP (anchor) signals relative to the 3D bin contact signals. To this end, we compared the irreproducible discovery rate (IDR) [18] (Section 2.7) to the HPRep results utilizing the highest read depth brain sample (RG1). This was accomplished by pairwise comparisons between the original ChIP-Seq data (IDR) or AND/XOR data (HPRep) and corresponding samples that had been downsampled to 80%, 60%, 40%, and 20% to the original depth. As expected, both IDR and HPRep metrics decreased with more aggressive downsampling, however, the effect on IDR, as measured by fraction of peaks passing a false discovery rate threshold of 5%, was far more pronounced. HPRep metrics were 0.97, 0.96, 0.93, and 0.88 compared to IDR of 0.80, 0.68, 0.24, and 0.06 at 80%, 60%, 40%, and 20% of the original depth, respectively. This effect difference suggests that 1D information does not dominate our results; if the HPRep results were merely a reflection of anchor similarity, we would expect a more consistent trend between the two experiments.

## 4. Discussion

Quantification of data reproducibility is critical to ensure scientific rigor, however, methods tailored for HiChIP and PLAC-Seq data are still lacking. Here, we propose HPRep, the first model-based approach to account for ChIP enrichment in measuring HP data reproducibility. Given the lack of HP specific tools, we compared HPRep to existing methods designed for Hi-C data, specifically HiCRep and HiC-Spector. Additionally, since our method, similar to HiCRep, relies on a weighted average of Pearson correlation coefficients, we also compared HPRep to the naïve Pearson correlation coefficient.

Our HPRep method, improving on existing Hi-C specific methods, was tailored to HP data for the measurement of reproducibility in two fundamental ways. First, HPRep was designed to accommodate the specific structure of HP data: while Hi-C data consist of contact frequencies among all bin pairs, HP data focuses on bin pairs where at least one bin overlaps with a ChIP-Seq peak for a protein of interest. This was different from the standard *n × n* symmetric Hi-C contract matrix. We focused on the data matrix on anchor bins, regions that overlapped with ChIP-Seq peaks, and pairs between bins within a specified window of these anchors as illustrated in Figure 1.

Second, HPRep fits a positive Poisson regression model to normalize HP-specific ChIP enrichment and uses the residuals as the normalized contact frequencies. It also analyzes bin pairs in the AND and XOR sets separately, effectively accounting for ChIP enrichment for the two different types of bin pairs.

Our results from mouse H3K4me3 PLAC-Seq data demonstrated very low variability in metrics between chromosomes (Figure 2), which is consistent with HiCRep (Supplementary Figure S3). In addition, we also compared HPRep with other existing methods using human H3K27ac HiChIP data from GM12878 and K562 cells as well as H3K4me3 PLAC-Seq data from four human brain cell types. Our results demonstrated the superior performance of HPRep, in terms of accurate clustering of samples from the human brain cell types, which was not achievable using HiCRep, although better clustering accuracy was observed when excluding bin pairs in the NOT set.

Future work involves exploring the potential of using this method to determine minimum per sample sequencing depth or maximum allowable (if any) differential depth across samples for accurate quantification of HP data reproducibility. We show that sample differentiation and expected clustering were robust to downsampling, but rigorous analysis needs to be performed in order to demonstrate practical use, as more high-depth HP data become available from more tissues, cell lines, or cell types. Additionally, we plan to examine the use of this general framework with capture Hi-C datasets including those targeting a relatively small number of loci identified from genome-wide association studies, and these genome-wide promoter capture Hi-C experiments. The use of pre-defined anchors by these methods suggests that the HPRep framework will be also applicable to these capture Hi-C methods, therefore these extensions are highly warranted but are beyond the scope of our current HPRep work. 

In terms of computational efficiency, for the human PLAC-Seq data consisting of 11 samples, tuning the smoothing parameter and determining all 55 pairwise reproducibility metrics for all 22 autosomal chromosomes took 1 h and 5 min using a single core on a 2.50 GHz Intel processor with 4GB of RAM. One can choose to apply HPRep to one chromosome for almost the same result. Using the same data, HPRep takes 35 min to perform tuning and analysis solely on chromosome 1 using the same single core.

## 5. Conclusions

Here, we present HPRep, a computationally efficient algorithm based on positive Poisson regression [13] and a stratified Pearson correlation [6]. Our comprehensive benchmark analyses of real HP datasets demonstrate that HPRep outperforms existing Hi-C reproducibility measurements.

## Data Availability

The data sources used in this manuscript are publicly available and described in Table S2.

## Supplementary Materials

The following are available online at https://www.mdpi.com/article/10.3390/cimb43020082/s1.
