# Supplementary material for: HPRep: Quantifying Reproducibility in HiChIP and PLAC-Seq Datasets
Source: Curr Issues Mol Biol. 2021 Sep 17;43(2):1156–70. doi: 10.3390/cimb43020082 (PMC8929028; doi:10.3390/cimb43020082)
Supplement: Supplementary file 1 [file cimb-43-00082-s001.zip › cimb-1368976-supplementary.pdf]

Supplementary Materials

HPRep: Quantifying reproducibility in HiChIP and PLAC-Seq datasets.

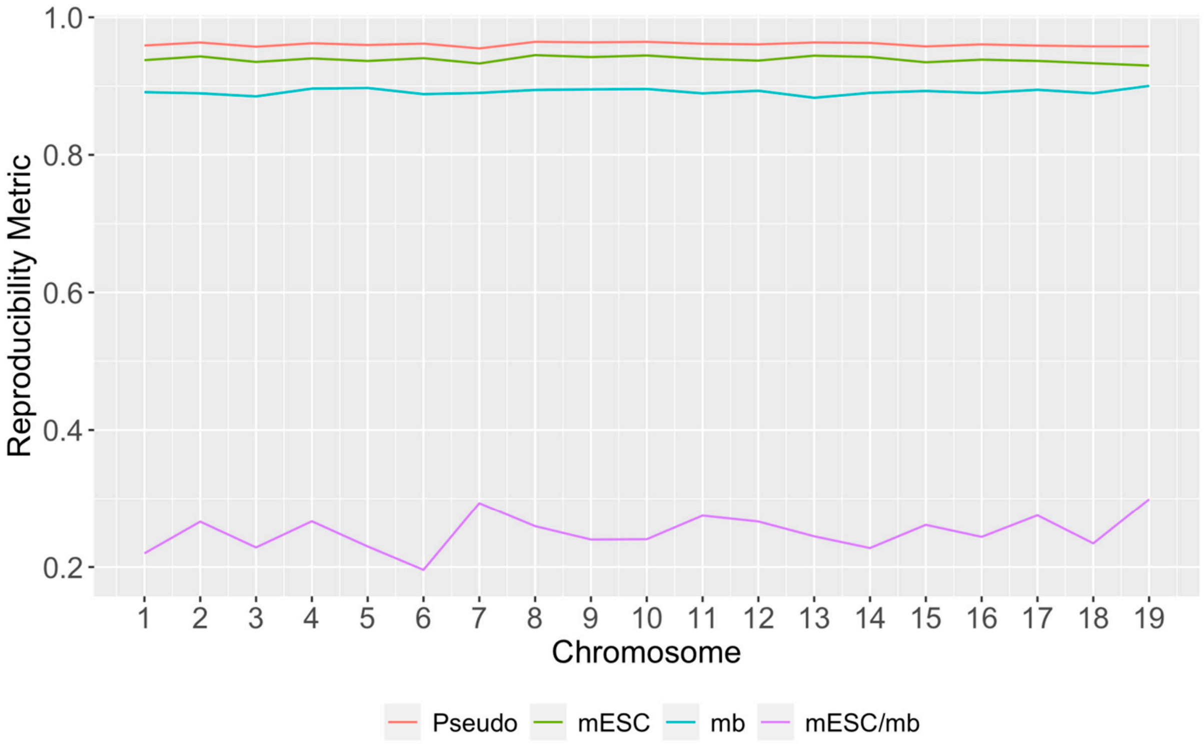

Figure S1. Reproducibility metric by chromosome. Metrics obtained applying HPRep to two mouse embryonic stem cell (mESC) and mouse brain (mb) tissues H3K4me3 PLAC-Seq samples. Pseudo replicates were generated from pooling mESC samples followed by random sampling via a binomial (p = 0.5) distribution. Cross sample results represent the mean of four cross-tissue pairings.

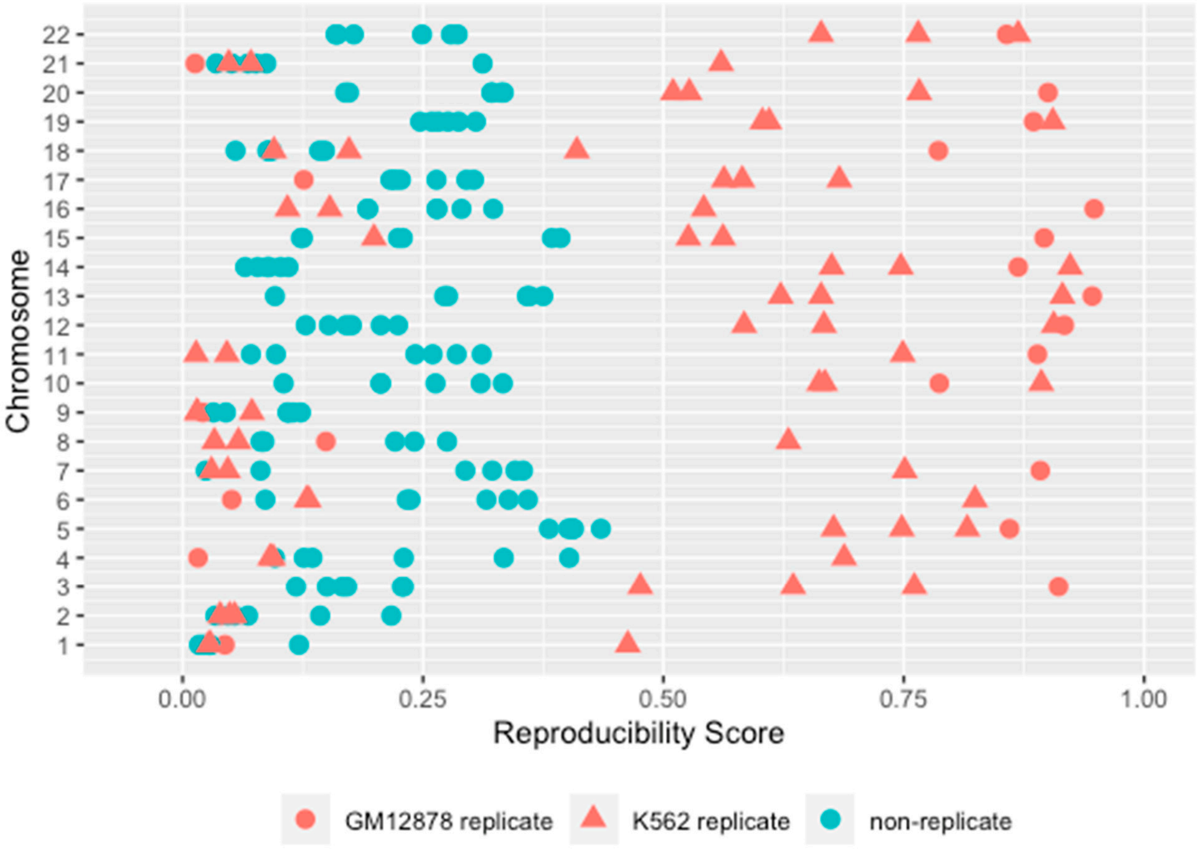

Figure S2. HiC-Spector results by chromosome. The plotted results clearly demonstrate the chromosome to chromosome variability we do not see with HiCRep or HPRep with these data. For example, the chromosome 22 results are as expected whereas the chromosome 21 results failed to distinguish between five of the six non-replicates and three of the four replicates.

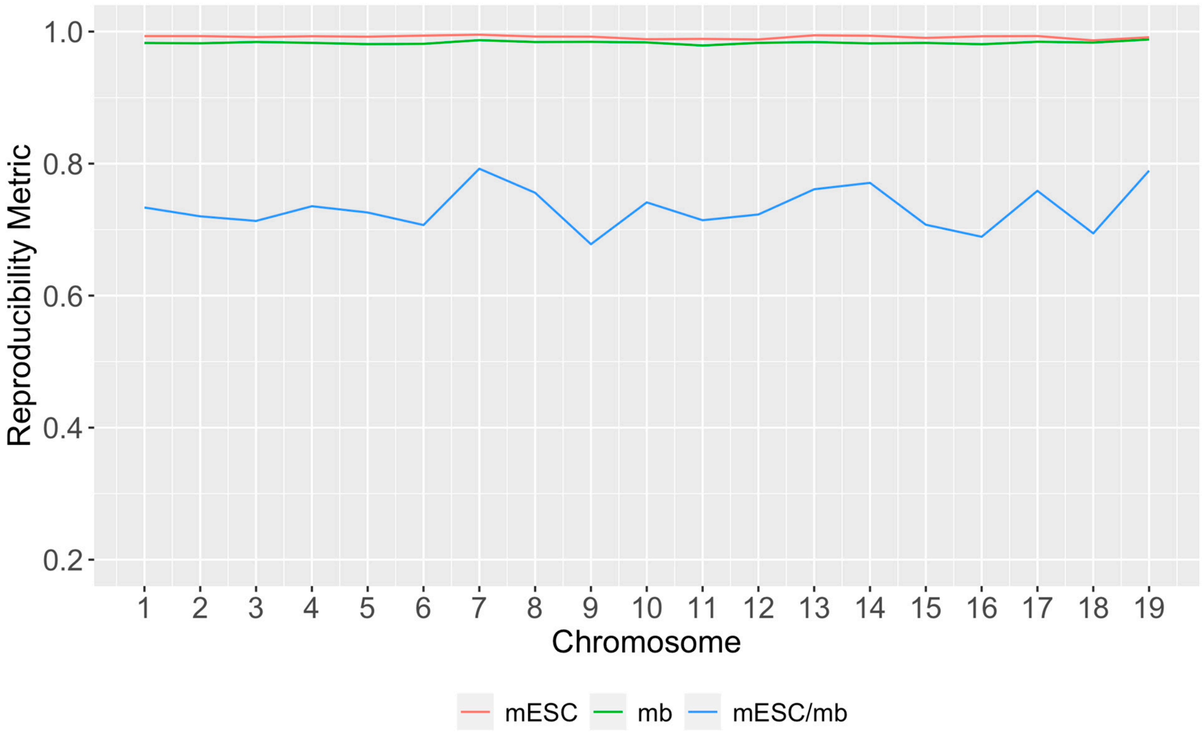

Figure S3. HiCRep reproducibility metric by chromosome. Metrics obtained applying HiCRep to two mouse embryonic stem cell (mESC) and mouse brain (mb) tissues H3K4me3 PLAC-Seq samples. Cross sample results represent the mean of four cross-tissue pairings.

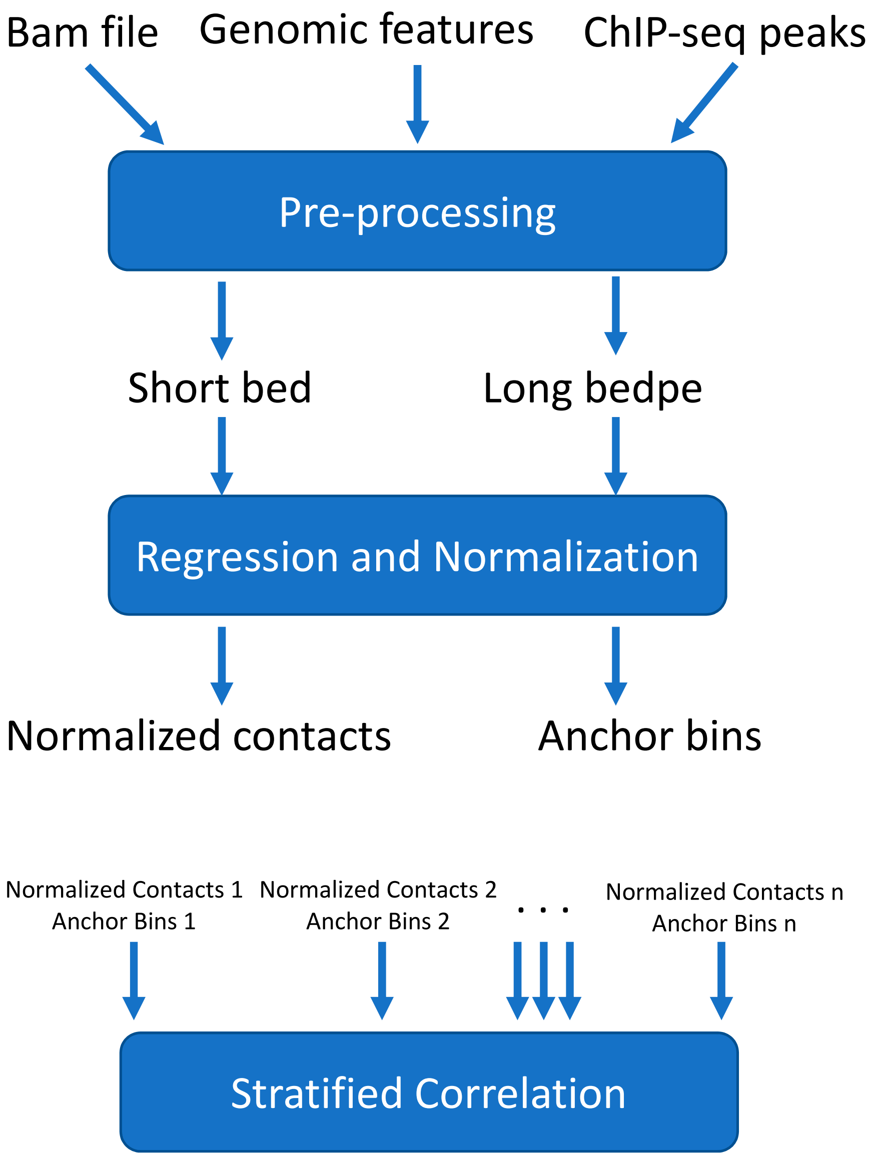

Figure S4. HPRep workflow.

Table S1. Number of effective eigenvectors in HiC-Spector analysis.

	Chromosome																					
	1	2	3	4	5	6	7	8	9	10	11	12	13	14	15	16	17	18	19	20	21	22
GM12878 replicate	18	19	20	18	20	19	20	20	18	19	20	20	20	19	20	20	19	19	20	20	16	19
Non-replicate 1	19	19	20	19	20	19	20	18	19	19	20	20	20	19	20	19	19	20	20	20	16	20
Non-replicate 2	18	19	20	19	20	19	18	20	19	20	18	20	20	19	19	19	19	19	20	20	16	20
Non-replicate 3	19	19	20	18	20	19	19	20	19	20	20	20	20	19	20	20	19	19	20	20	16	20
Non-replicate 4	17	19	20	18	20	19	20	18	19	19	20	20	19	20	20	19	19	18	20	20	18	20
Non-replicate 5	17	20	20	18	20	18	18	20	19	19	18	20	20	20	19	19	19	20	20	20	18	20
Non-replicate 6	17	18	20	18	20	19	19	20	19	19	20	20	20	19	20	20	19	20	20	20	18	20
K562 replicate 1	16	19	20	19	20	19	19	20	19	19	18	20	19	20	18	19	20	18	20	20	19	19
K562 replicate 2	19	19	20	19	20	19	19	20	20	19	20	20	19	19	20	20	20	18	20	20	19	19
K562 replicate 3	18	18	20	19	20	19	19	20	19	20	19	20	20	19	18	19	20	18	20	20	18	20

Table 1 displays the number of effective eigenvectors used in the analysis of the cohesion HiChIP dataset using NOT data from Figure 3. In conjunction with Supplementary Figure S1, it illustrates how utilization of fewer than 20 eigenvectors affects the outcome with respect to the expected values. For example, the analyses of chromosomes 3 and 12 utilized 20 eigenvectors for all 10 pairs and the biological replicates all had higher metrics than the non-replicates. This was compared with the results for chromosomes 1 and 2, whose analyses utilized almost exclusively fewer than 20 eigenvectors and the biological and non-replicates were not distinguished.

Table S2. Data sources.

Data Description	Reference (PMID)	GEO accession number or other sources
mESC H3K4me3 PLAC-Seq	30986246	GSE119663
Mouse brain H3K4me3 PLAC-Seq	31068695	GSE127995
GM12878 H3K27ac HiChIP	28945252	GSE101498
K562 H3K27ac HiChIP	28945252	GSE101498
Human brain H3K4me3 PLAC-Seq	33057195	Neuroscience Multi-Omic Archive (NeMO Archive) under controlled access
